# Identity Statuses throughout Adolescence and Emerging Adulthood: A Large-Scale Study into Gender, Age, and Contextual Differences

**DOI:** 10.5334/pb.348

**Published:** 2017-04-04

**Authors:** Margaux Verschueren, Jessica Rassart, Laurence Claes, Philip Moons, Koen Luyckx

**Affiliations:** 1 Faculty of Psychology and Educational Sciences, KU Leuven, BE; 2 Faculty of Medicine and Health Sciences (CAPRI), University of Antwerp, BE; 3 Department of Public Health and Primary Care, Academic Center for Nursing and Midwifery, KU Leuven, Leuven, BE; 4 Institute of Health and Care Sciences, University of Gothenburg, SE

**Keywords:** identity, exploration, commitment, adolescence, emerging adulthood

## Abstract

Identity formation constitutes a core developmental task during adolescence and emerging adulthood. However, it remains unclear how identity formation may vary across age, gender, and context (education vs. employment) in these developmental periods. The present study used a recently developed model to examine identity statuses or types in a sample of 7,906 Flemish individuals (14–30 years old; 64% female). As expected, achievement, foreclosure, moratorium, carefree diffusion, troubled diffusion, and an undifferentiated status emerged through cluster analysis. Women were overrepresented in the moratorium status (characterized by high exploration), whereas men were mainly situated in foreclosure and carefree diffusion statuses (both characterized by low exploration, but individuals in foreclosure having strong identity commitments as well). Individuals in the carefree and troubled diffusion statuses, which represent the least adaptive statuses, were youngest. High school students were overrepresented in the diffusion statuses and college students were mostly present in achievement (representing the most mature status) and moratorium. Finally, employed individuals were overrepresented in foreclosure, whereas unemployed individuals were mainly situated in troubled diffusion. In sum, the present study systematically examined relationships between empirically-identified identity statuses and socio-demographic variables in a large-scale sample, generating important information on age, gender, and contextual differences in identity.

## Introduction

Although identity formation represents the core developmental task of adolescence (Erikson, 1968), the identity search may continue into the twenties. Arnett (2000) introduced the concept of emerging adulthood, a period ranging from the late teens through the twenties, characterized by life changes and identity exploration. As emerging adults may struggle with their newly adopted roles, this phase should not be overlooked in identity research. Based on Erikson (1968), J. Marcia (1966) described two processes at the heart of this identity task: exploration (i.e., actively questioning different alternatives) and commitment (i.e., making life choices). Based on these processes, Marcia derived four statuses: achievement (commitment following exploration), foreclosure (commitment without exploration), moratorium (exploration without commitment), and diffusion (no commitment or exploration).

These identity processes have been refined by Luyckx, Schwartz, Berzonsky, et al. (2008) into two commitment (commitment making and identification with commitment) and three exploration processes (exploration in breadth, exploration in depth, and ruminative exploration). Individuals often start the identity process by actively exploring different options (*exploration in breadth*) before making decisions (*commitment making*). Subsequently, they may re-evaluate these commitments based on personal beliefs and values (*exploration in depth*) through which these choices may or may not become integrated into their sense of self (*identification with commitment*). However, exploration may not always be helpful towards identity formation. Continuously exploring alternatives and revisiting the same identity questions (*ruminative exploration*) may be accompanied by worry, indecisiveness, and distress and may hinder identity development (Luyckx, Schwartz, Berzonsky, et al., 2008).

When combining these five identity processes through cluster analysis, six identity statuses (further extending Marcia’s statuses) have been identified: achievement, foreclosure, moratorium, carefree diffusion, troubled diffusion, and an undifferentiated cluster (Luyckx, Schwartz, Goossens, Beyers, & Missotten, 2011). Identity achievement and foreclosure, characterized by high scores on commitment processes and low scores on ruminative exploration, represent adaptive statuses. Individuals in achievement score higher on exploration in breadth and in depth as compared to individuals in foreclosure, testifying to their open and information-oriented nature. The moratorium status is characterized by high scores on all exploration processes, but low to moderate scores on commitment processes. These individuals are still exploring, but have not yet made strong commitments. Individuals in both diffusion statuses score relatively low on all adaptive identity processes. In troubled diffusion, characterized by high levels of ruminative exploration, individuals are unable to take proactive steps in identity development. In contrast, individuals in carefree diffusion seem rather unbothered by their lack of pro-active identity work and, consequently, also do not ruminate about identity issues. Finally, the undifferentiated status represents individuals who score moderate on all processes.

Previous research, including mostly student samples, has focused on how these six statuses relate to psychosocial correlates. Individuals in foreclosure and achievement seem to show the most adaptive general functioning, as they score high on self-esteem and well-being (Crocetti, Luyckx, Scrignaro, & Sica, 2011; Schwartz et al., 2011). Conversely, individuals in moratorium and diffusion seem to experience more depressive symptoms, anxiety, and aggressive behavior. Especially individuals in troubled diffusion (characterized by high levels of ruminative exploration) show the least adaptive psychosocial functioning (Crocetti et al., 2011; Lillevoll, Kroger, & Martinussen, 2013; Schwartz et al., 2011). Hence, the development of a clear personal identity seems to protect individuals against a broad range of clinical symptoms. The present study extends this research line by (1) identifying identity statuses in a large, socio-demographically diverse sample, and (2) examining how these statuses differ on these socio-demographic variables (gender, age, educational-employment context). First, we conducted cluster-analysis on the five identity processes and expected the following statuses to emerge: achievement (high on commitment and adaptive exploration processes and low on ruminative exploration), foreclosure (high on commitment processes and low on all three exploration processes), moratorium (high on all three exploration processes and low to moderate on commitment processes), carefree diffusion (low on all identity processes), troubled diffusion (low on all identity processes, but high on ruminative exploration), and undifferentiated (intermediate on all identity processes).

Second, we examined whether individuals were distributed differentially across these statuses depending on gender, age, and educational-employment context. With respect to gender, few systematic gender differences in identity status have been reported in earlier research (Kroger, 1997). However, in more recent studies, more gender differences have been described. Women have been found to be overrepresented in achievement, whereas men were situated more in carefree diffusion (Meeus, Van De Schoot, Keijsers, Schwartz, & Branje, 2010; Schwartz et al., 2011). These results are in line with research on identity processing styles (i.e., social-cognitive strategies that are used to construct a sense of identity) demonstrating that men are more likely to avoid dealing with identity conflicts and to adopt a present-oriented, hedonistic perspective (Berzonsky, 2008, 2011; Soenens, Berzonsky, Vansteenkiste, Beyers, & Goossens, 2005). The reason for such a rather avoidant attitude towards identity issues in men is not entirely clear but a possible explanation may lay in sex-role socialization processes and parenting behaviors (with more freedom and less supervision being granted for boys than for girls; Berzonsky, 2008; Berzonsky & Kinney, 2008). Other studies have found women to score higher on exploration in depth and ruminative exploration (Crocetti et al., 2011; Luyckx, Gandhi, Bijttebier, & Claes, 2015; Luyckx, Schwartz, Berzonsky, et al., 2008). These findings could be explained by the fact that women tend to be more indecisive in nature and are more information-oriented when being faced with a challenge (Rassin & Muris, 2005). Moreover, women have been found to ruminate more than men and are especially susceptible to anxiety-related cognitive factors (Craske, 2003; Johnson & Whisman, 2013). Consequently, we expected women to be overrepresented in achievement and moratorium, both characterized by exploration, as well as in troubled diffusion, characterized by ruminative exploration. We expected men to be overrepresented in carefree diffusion.

With respect to age, we expected, in line with the identity status continuum in which a progressive identity development from diffusion to achievement is described with increasing age (Kroger, Martinussen, & Marcia, 2010; Meeus, 2001), individuals in diffusion being youngest and individuals in achievement being oldest. Relatedly, we expected high school students to be overrepresented in the diffusion statuses, as they may be at the starting point of identity development and may be relatively confused about their position in life. In contrast, emerging adulthood is considered a transitional phase to young adulthood, characterized by elevated levels of exploration and a sense of wide-open possibilities (Arnett, 2004). However, previous research has indicated substantial differences between the college and work context as well. The college setting offers a broad range of ideological perspectives, educational possibilities, and alternative worldviews, which presents the best opportunity for self-exploration (Munro & Adams, 1977). Students may try out different life paths, in order to evaluate future lifestyles and commitments to which they wish to adhere. In contrast, emerging adults who are already at work seem to handle identity issues in a different manner. The concrete nature of the work setting seems to limit future possibilities, as the current occupation already indicates a certain direction in life (Buhl, 2007; Munro & Adams, 1977). Furthermore, employment has been related to experiencing a greater sense of adulthood, which was partially mediated by having made strong life commitments (Luyckx, Schwartz, Goossens, & Pollock, 2008). Consequently, we expected college students to be overrepresented in moratorium and employed individuals to be more situated in foreclosure and achievement. Finally, as unemployment may threaten an individual’s identity and has been related to a loss of meaning in life (McKee-Ryan, Song, Wanberg, & Kinicki, 2005; Schöb, 2013), we expected unemployed individuals to be overrepresented in the diffusion statuses.

## Methods

### Participants and procedure

Nineteen samples of individuals aged 14 to 30 years (*M* = 18.28; *SD* = 3.55) collected between 2007 and 2015 in Flanders (Belgium) were combined, totalling to 7,906 participants (64% female): 4,357 were in high school, 2,224 were college or university students, 1,092 were employed, and 32 were unemployed. Table 1 displays an overview of demographic characteristics. A total of 62% high school students followed the academic track, and 38% followed the technical or the vocational track. College students were mostly from the KU Leuven and, although the majority was from the Faculty of Psychology and Educational Sciences, students from other majors were also included. For the (un)employed individuals, questionnaires were distributed in different work settings, such as schools, hospitals, and private companies, or via e-mail and social media (e.g., Facebook). All studies were approved by an authorized ethical commission and participants gave informed consent.

**Table 1:** Demographic Characteristics of the 19 Samples.

	*N*	% female	*M* (*SD*) age	Age range	Sample description

Sample 1	208	79	18.18 (1.39)	17–26	College students
Sample 2	369	78	18.25 (1.27)	16–30	College students
Sample 3	371	65	23.28 (3.21)	17–30	College students (54%); employed individuals (45%)
Sample 4	345	70	23.89 (2.85)	18–30	College students (41%); employed individuals (59%)
Sample 5	353	78	18.50 (1.02)	17–28	College students
Sample 6	342	40	18.29 (0.60)	17–21	High school students
Sample 7	456	84	18.36 (1.35)	17–30	College students
Sample 8	600	52	15.70 (1.30)	14–20	High school students
Sample 9	193	82	25.73 (2.30)	21–30	Employed individuals
Sample 10	249	63	16.49 (0.68)	15–19	High school students
Sample 11	407	84	18.35 (1.41)	17–29	College students
Sample 12	567	51	15.80 (1.02)	14–18	High school students
Sample 13	1,388	64	15.72 (1.19)	14–18	High school students
Sample 14	404	49	16.29 (1.04)	14–19	High school students
Sample 15	564	61	16.14 (1.41)	14–21	High school students
Sample 16	279	57	22.54 (3.54)	18–30	College students (58%); employed individuals (42%)
Sample 17	243	50	15.53 (1.17)	14–19	High school students
Sample 18	381	38	22.95 (2.39)	18–30	College students (61%); employed individuals (39%)
Sample 19	201	56	25.16 (2.58)	20–30	College students (19%); employed individuals (81%)

### Measure

**Identity processes.** Participants completed the Dimensions of Identity Development Scale (DIDS; Luyckx, Schwartz, Berzonsky, et al., 2008), which was developed in Dutch and provides reliable scores with a clear factor structure (Luyckx et al., 2011). Each of the identity processes was measured by five items, to be answered on a 5-point Likert-type rating scale, ranging from 1 (*Strongly disagree*) to 5 (*Strongly agree*). Sample items include “I have decided on the direction I want to follow in my life” (commitment making), “I sense that the direction I want to take in my life will really suit me” (identification with commitment), “I regularly think over a number of different plans for the future” (exploration in breadth), “I regularly talk with other people about the plans for the future I have made for myself” (exploration in depth), and “It is hard for me to stop thinking about the direction I want to follow in my life” (ruminative exploration). Cronbach’s alphas were .90, .86, .84, .80, and .83, respectively.

## Results

**Figure 1:**
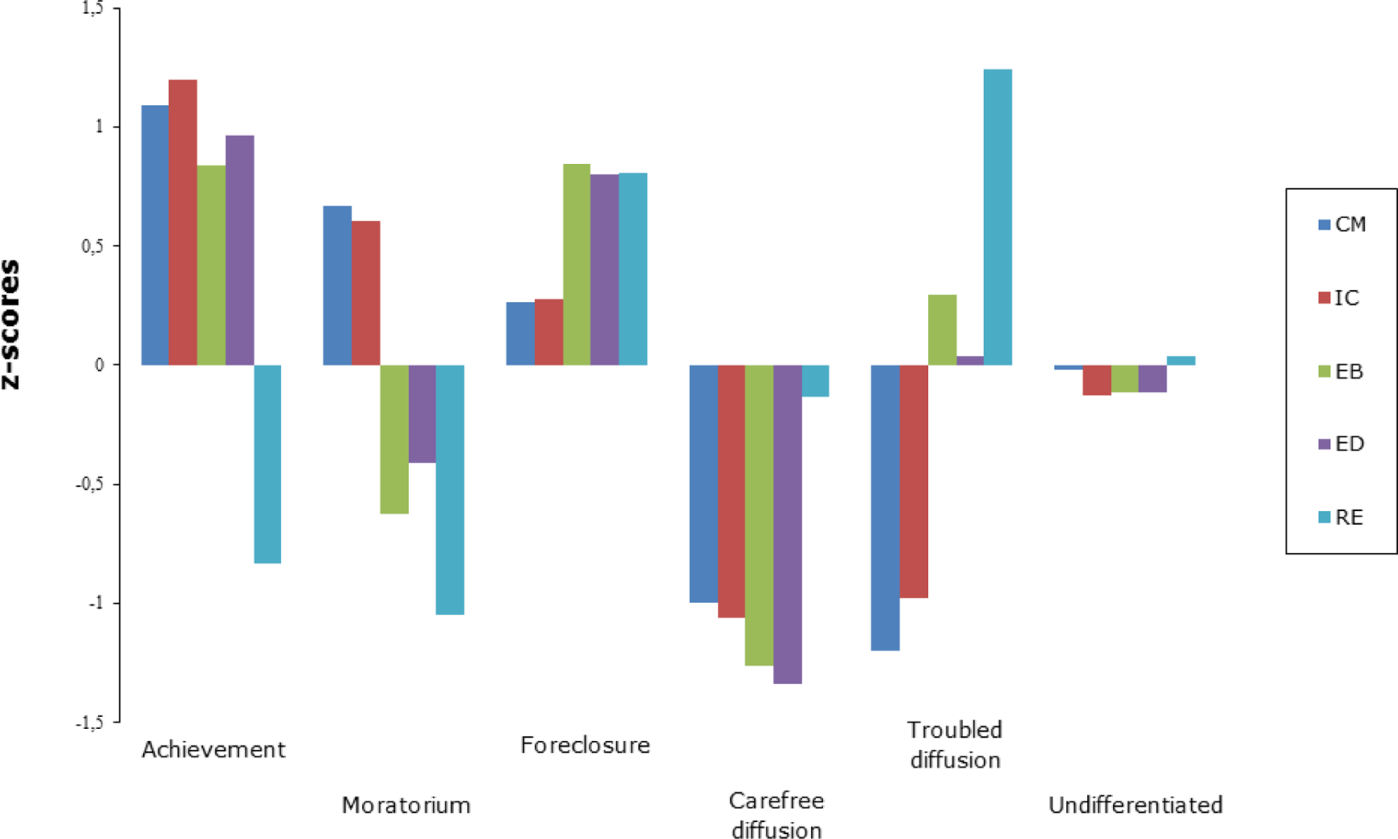
Standardized *Z*-scores for the identity processes for the final six-cluster solution. CM = commitment making; IC = identification with commitment; EB = exploration in breadth; ED = exploration in depth; RE = ruminative exploration.

Cluster analysis on the identity processes was conducted using a two-step procedure (Gore, 2000). Prior to all analyses, 116 univariate and multivariate (individuals with high Mahalanobis distance values) outliers were removed and four- to six-cluster solutions were evaluated. First, a hierarchical cluster analysis was carried out using Ward’s method based on squared Euclidian distances. Second, these initial cluster centres were used as non-random starting points in an iterative *k*-means clustering procedure. Six clusters were retained based on interpretability, parsimony, and explanatory power, explaining between 55% and 62% of the variance in identity processes (see Figure 1 in which the Y-axis represents z-scores: 0.2 SD is a small effect, 0.5 SD a medium effect, and 0.8 SD a large effect; Cohen, 1988).

As expected, individuals in achievement scored high on commitment processes, exploration in breadth and in depth, and low on ruminative exploration (*N* = 1,247; 16%). Individuals in foreclosure scored moderately high to high on commitment processes and moderately low to low on all exploration processes (*N =* 1,156; 15%). Individuals in moratorium scored moderate on both commitment processes, and high on the exploration processes (*N* = 1,246; 16%). Individuals in carefree diffusion scored low on all identity processes, except for a moderate score on ruminative exploration (*N* = 999; 13%). Individuals in troubled diffusion scored low on both commitment processes, moderate on exploration in breadth and in depth, and high on ruminative exploration (*N* = 1,051; 14%). Individuals in the undifferentiated cluster scored moderate on all processes (*N* = 2,027; 26%).

With respect to age, univariate analysis of variance pointed to a significant age effect *F*(5, 7720) = 40.42, *p* < .001, partial η² = .03), with individuals in carefree diffusion (*M* = 17.23; *SD* = 3.25) being significantly younger (with all *p*s < .001) than individuals in the remaining clusters. Further, individuals in troubled diffusion (*M* = 17.99; *SD* = 3.22) and the undifferentiated cluster (*M* = 18.09; *SD* = 3.37) were significantly younger than individuals in moratorium (*M* = 18.67; *SD* = 3.37), foreclosure (*M* = 19.13; *SD* = 4.31), and achievement (*M* = 18.68; *SD* = 3.50). Finally, individuals in foreclosure were significantly older than individuals in all other statuses.

To investigate the associations between identity statuses and gender and context (high school, college/university, employed, unemployed), χ²-analyses were conducted. Standardized residuals exceeding |2| were indicative of a significant discrepancy between observed and expected frequencies in the respective cell. As displayed in Table 2, males were relatively overrepresented in foreclosure and carefree diffusion and underrepresented in moratorium, whereas females were overrepresented in moratorium and underrepresented in foreclosure and carefree diffusion.

**Table 2:** Cross-Tabulation of Six Identity Clusters by Gender.

Identity clusters	Male	Female	*Total*

Achievement	425 (–0.6) 32%	822 (0.4) 68%	1,247
Foreclosure	**459 (2.7) 40%**	**695 (**–**2.0) 60%**	1,154
Moratorium	**361 (**–**3.6) 29%**	**883 (2.7) 71%**	1,244
Carefree Diffusion	**420 (3.7) 42%**	**578 (**–**2.7) 58%**	998
Troubled Diffusion	340 (–1.5) 32%	710 (1.1) 68%	1,050
Undifferentiated	704 (–0.3) 35%	1,321 (0.2) 65%	2,025
*Total*	2,709 35%	5,009 65%	7,718

*Note*. Standardized residuals within parentheses. Percentages represent the proportion of males and females within each identity cluster. Cells in bold exceed a standardized residual of |2|.

Next, as displayed in Table 3, high school students were relatively overrepresented in carefree and troubled diffusion and underrepresented in achievement, foreclosure, and moratorium. College and university students were overrepresented in achievement and moratorium and underrepresented in foreclosure and carefree diffusion. Employed individuals were overrepresented in foreclosure and underrepresented in carefree and troubled diffusion and the undifferentiated cluster. Finally, unemployed individuals were underrepresented in foreclosure and overrepresented in troubled diffusion.

**Table 3:** Cross-Tabulation of Six Identity Clusters by Context.

Identity clusters	High school	College/University	Employment	Unemployment	*Total*

Achievement	**618 (–2.5) 50%**	**449 (3.3) 36%**	117 (0.2) 14%	3 (–1.0) 0%	1,247
Foreclosure	**576 (–2.3) 50%**	**299 (–3.1) 26%**	**281 (9.4) 24%**	**0 (–2.2) 0%**	1,156
Moratorium	**594 (–3.4) 48%**	**469 (4.3) 38%**	175 (0.1) 14%	8 (1.2) 1%	1,246
Carefree Diffusion	**692 (6.2) 69%**	**215 (–5.3) 22%**	**90 (–4.2) 9%**	2 (–1.1) 0%	999
Troubled Diffusion	**639 (2.7) 61%**	289 (–1.9) 28%	**111 (–3.0) 11%**	**11 (3.2) 1%**	1,050
Undifferentiated	1,111 (0.0) 55%	662 (1.5) 33%	**246 (–2.2) 12%**	8 (–0.1) 0%	2,027
*Total*	4,230 55%	2,383 31%	1,080 14%	32 0%	7,725

*Note*. Standardized residuals within parentheses. Percentages represent the proportion of individuals in the different contexts within each identity cluster. Cells in bold exceed a standardized residual of |2|.

## Discussion

The present study examined identity statuses in a large sample of Flemish adolescents and emerging adults. We relied on a recently developed identity model which distinguishes among two commitment processes (commitment making and identification with commitment), two pro-active exploration processes (exploration in breadth and exploration in depth), and a ruminative exploration process (Luyckx, Schwartz, Berzonsky, et al., 2008). The six clusters obtained (achievement, foreclosure, moratorium, troubled diffusion, carefree diffusion, undifferentiated) were as expected and extended and refined Marcia’s (1980) seminal identity status paradigm. Important differences among the identity statuses were found for age, gender, and educational-employment context. Hence, the present study generated important information that can inform developmental theory about identity formation across the teens and twenties.

Systematic differences among these empirically identified statuses were identified for gender, age, and educational-employment context. In line with expectations, women were overrepresented in moratorium, a primarily exploration-based status, which corroborates women’s higher levels of being information-oriented but also their indecisive and ruminative nature (Johnson & Whisman, 2013; Rassin & Muris, 2005). In contrast, men were overrepresented in carefree diffusion and foreclosure, both characterized by low scores on all exploration processes. Hence, men seem generally less inclined to take pro-active steps in identity exploration and, even when making important life decisions, they seem to explore less as compared to women. These results support earlier findings of men being more likely to adopt the diffuse-avoidant identity style, in which pro-active identity work is avoided (Berzonsky, 2008, 2011; Soenens et al., 2005). The overrepresentation of men in foreclosure is consistent with a study by Archer (1989), in which individuation, task-orientation, and agency are discussed as typical male sex roles in our society. Hence, these characteristics could stimulate the making of identity commitments in men.

With respect to age, our results were in line with the identity status continuum following a progressive identity development (Kroger et al., 2010; Meeus, 2001). Individuals in the diffusion and in the undifferentiated clusters were significantly younger than the remaining statuses, with individuals in carefree diffusion being youngest. Similarly, we found high school students to be overrepresented in the diffusion statuses, illustrating the confusion and uncertainty they may experience about their position in early life. In contrast, college students were more situated in moratorium, primarily pointing to the fact they are actively exploring different life options. These results support the idea that the college context allows for a broad range of future possibilities, worldviews, and identity exploration (Arnett, 2000; Luyckx, Goossens, & Soenens, 2006; Munro & Adams, 1977).

Additionally, and contrary to expectations, college students rather than employed individuals were overrepresented in the achievement status. This result may be due to differences in exploration between the two contexts. College students seem to explore more in a pro-active and organized manner (e.g., trying out different college majors), while employed emerging adults often express a sense of meandering, in which they rather “fall into” their job (Arnett, 2004). The overrepresentation of college students in achievement may be indicative of individuals that have found their true calling after having explored various identity alternatives during the college years. Similarly, a study by Luyckx et al. (2006) found an increase in both exploration of identity alternatives and commitment making during the college years. Hence, the college setting seems not only to stimulate exploration, but also may motivate individuals to commit to important life decisions. Conversely, when compared to the college context, the work setting seems to offer less opportunities for pro-active identity exploration, as employment guides future directions and may even limit future possibilities (Arnett, 2004; Buhl, 2007). Our results indicate an overrepresentation of employed individuals in foreclosure, again indicative of the fact that employed individuals may adhere to certain life choices more frequently without a profound exploration process (Luyckx, Schwartz, Goossens, et al., 2008; Yoder, 2000).

Finally, we found unemployed individuals to be overrepresented in troubled diffusion and underrepresented in foreclosure. Hence, these individuals seem to experience problems in committing to life decisions and tend to explore more in a ruminative manner. As occupational commitments comprise a core element of one’s personal identity (Erikson, 1968), unemployment may be perceived as a loss of control and meaning in life (McKee-Ryan et al., 2005; Price, Friedland, & Vinokur, 1998). These findings corroborate with the idea that unemployment may threaten an individual’s identity, especially during the transition to adulthood (Danielsen, Lorem, & Kroger, 2000; Schöb, 2013).

The present study was characterized by some limitations. First, the cross-sectional design prevents us from drawing conclusions about developmental changes in identity statuses. Long-term longitudinal research could offer more insight. Second, whereas self-report questionnaires remain the optimal way to investigate internal processes such as identity, including alternative methods to gather information (e.g., reports by family members or interviews) could help in validating our findings. Third, our sample only comprised a limited number of unemployed individuals, due to which our results may not be representative of the Flemish demography. In future research, including a larger number of unemployed individuals is recommended. Finally, the present study only focused on a specific identity model, beyond which the present findings cannot be generalized. Future research including other methodologies (e.g., narrative viewpoint on identity) could provide interesting results.

Despite these limitations, the present study generated important information on systematic differences in identity statuses for several socio-demographic variables (gender, age, and educational-employment context) and, hence, may provide a knowledge base for future research.
