# Correction: Identity Statuses Throughout Adolescence and Emerging Adulthood: A Large-scale Study into Gender, Age, and Contextual Differences

**DOI:** 10.5334/pb.440

**Published:** 2018-02-14

**Authors:** Margaux Verschueren, Jessica Rassart, Laurence Claes, Philip Moons, Koen Luyckx

**Affiliations:** 1 Faculty of Psychology and Educational Sciences, KU Leuven, BE; 2 Faculty of Medicine and Health Sciences (CAPRI), University of Antwerp, BE; 3 Department of Public Health and Primary Care, Academic Center for Nursing and Midwifery, KU Leuven, Leuven, BE; 4 Institute of Health and Care Sciences, University of Gothenburg, SE

**Keywords:** identity, exploration, commitment, adolescence, emerging adulthood

## Abstract

This article details a correction to the article: Verschueren, M., et al., (2017). Identity Statuses throughout Adolescence and Emerging Adulthood: A Large-Scale Study into Gender, Age, and Contextual Differences. *Psychologica Belgica*. 57(1), pp. 32–42. DOI: https://doi.org/10.5334/pb.348

## Correction

The original Figure 1 was incorrectly labelled in the published version of Verschueren (2017). The correct Figure 1 is below, with the labels ‘foreclosure’ and ‘moratorium’ switched to their correct positions.

**Figure 1:**
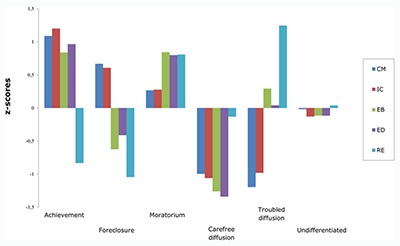
Standardized z-scores for the identity processes for the final six-cluster solution. CM = commitment making; IC = identification with commitment; EB = exploration in breadth; ED = exploration in depth; RE = ruminative exploration.
